# Tocolytic therapy for preterm premature rupture of membranes: a systematic review and meta-analysis

**DOI:** 10.61622/rbgo/2026rbgo33

**Published:** 2026-09-23

**Authors:** Luisa Melo Lucas, Ana Clara Felix de Farias Santos, Pedro Henrique Costa Matos da Silva

**Affiliations:** 1 Centro Universitário Alfredo Nasser Aparecida de Goiânia GO Brazil Centro Universitário Alfredo Nasser, Aparecida de Goiânia, GO, Brazil.; 2 Universidade Cidade de São Paulo São Paulo SP Brazil Universidade Cidade de São Paulo, São Paulo, SP, Brazil.; 3 Universidade Federal de Goiás Goiânia GO Brazil Universidade Federal de Goiás, Goiânia, GO, Brazil.

**Keywords:** Membrane premature rupture, Fetal membranes, Tocolysis, Preterm prelabor rupture of membranes

## Abstract

**Objective:**

To evaluate the effects of tocolytic therapy on maternal and neonatal outcomes in women with preterm prelabor rupture of membranes (PPROM) compared with no tocolysis, based on evidence from randomized controlled trials.

**Data sources:**

A systematic search was performed in MEDLINE, Embase, and the Cochrane Central Register of Controlled Trials (CENTRAL) from database inception to July 2024. The search strategy included controlled vocabulary terms and free-text terms related to PPROM and tocolysis.

**Study selection:**

Randomized controlled trials involving women with PPROM that compared any tocolytic therapy with no tocolysis or standard care were eligible for inclusion. Studies were selected independently by three reviewers, and disagreements were resolved by consensus.

**Data extraction and synthesis:**

Two reviewers independently extracted data using a standardized form. Risk of bias was assessed using the Cochrane Risk of Bias 2 tool. Dichotomous outcomes were summarized as odds ratios (ORs) with 95% confidence intervals (CIs) using a random-effects model. Heterogeneity was assessed using the I^2^ statistic. Analyses were performed using Review Manager (RevMan) version 5.4. The outcomes assessed included neonatal mortality, neonatal intensive care unit admission, necrotizing enterocolitis, intraventricular hemorrhage, and neonatal sepsis.

**Results:**

Four comparative studies, involving 372 participants including two randomized controlled trials and two non-randomized studies. Compared with no tocolysis or standard care, tocolytic therapy was not associated with a significant reduction in neonatal mortality (OR: 2.02; 95% CI: 0.51–8.01), neonatal intensive care unit admission (OR: 0.49; 95% CI: 0.02–14.57), necrotizing enterocolitis (OR: 1.35; 95% CI: 0.38–4.72), intraventricular hemorrhage (OR: 0.96; 95% CI: 0.01–138.63), or sepsis (OR: 1.18; 95% CI: 0.25–5.54).

**Conclusion:**

In women with PPROM, current evidence from randomized controlled trials does not demonstrate a clear neonatal benefit associated with tocolytic therapy compared with no tocolysis or standard care. Given the low certainty of evidence and the limited number of available studies, the routine use of tocolysis in this population remains uncertain and should be individualized until further high-quality randomized trials are available.

**Prospero registry:** #CRD420251043906

## Introduction

Preterm premature rupture of membranes (PPROM) is defined as the rupture of fetal membranes before 37 weeks of gestation and prior to the onset of labor. Diagnosis typically involves a speculum examination, revealing sudden fluid leakage from the cervix, characterized by a hyaline or yellowish appearance. Additionally, ultrasonography may assist in confirming oligohydramnios, particularly in cases of inconclusive clinical evaluation.^(1,2)^ PPROM has a multifactorial etiology, with factors such as alterations in vaginal microbiota, inflammation, weakened membranes, cervical insufficiency, and bleeding contributing to its occurrence. Major risk factors include a history of PPROM, genital infections, low socioeconomic status, vaginal bleeding, uterine overdistension, and smoking.^(1,2)^

PROM can cause severe maternal and neonatal complications, including perinatal morbidity and mortality, predominantly due to preterm delivery. PROM is responsible for approximately one-third of all preterm births. Significant neonatal morbidities include respiratory distress syndrome, intraventricular hemorrhage, necrotizing enterocolitis, and infections. Additional complications include premature placental abruption, umbilical cord prolapse, and abnormal fetal heart rate patterns.^(1,2)^

Maternal health concerns involve infections such as chorioamnionitis, endometritis, and bacteremia. Following membrane rupture, the risk of preterm labor increases significantly, with most women delivering within one week under conservative management. Protocols exist to enhance neonatal outcomes by optimizing newborn recovery chances and minimizing prematurity-related risks.^(3-5)^

Management of PPROM remains a controversial issue in perinatal medicine. Therapeutic decisions depend on multiple factors, including gestational age, maternal-fetal infections, presence of labor, fetal presentation and heart rate, uterine contractions, expected fetal lung maturity, cervical condition, and available neonatal support. Key management decisions include the choice between expectant management and active intervention, the use of tocolytic agents, antibiotic therapy duration, optimal corticosteroid timing, and infection detection strategies. An important component of clinical decision-making involves determining the ideal timing for delivery.^(4-6)^

Expectant management encompasses fetal monitoring, corticosteroid administration for pulmonary maturation, screening for infections, and prophylactic antibiotic treatment for seven days. Certain practices, such as supplemental progesterone, tissue sealants, and amnioinfusion, remain scientifically unvalidated. Immediate delivery is indicated upon suspicion of intrauterine infection, fetal distress, or placental abruption.^(7,8)^

Tocolysis is frequently employed as a therapeutic approach in cases of imminent preterm labor. Its primary objective is to delay delivery, allowing completion of corticosteroid administration to enhance fetal pulmonary maturation or enabling maternal transfer to a specialized high-complexity care center. Thus, tocolysis can significantly influence maternal and neonatal outcomes associated with preterm labor complications.^(9-12)^

Tocolysis remains a controversial intervention in women with preterm prelabor rupture of membranes (PPROM). Neonatal morbidity and mortality are strongly influenced by gestational age at delivery, and clinical management should carefully balance potential benefits and risks, particularly the risk of maternal and neonatal infectious complications. Further evidence is needed to clarify the impact of tocolytic therapy on clinically relevant neonatal outcomes.^(13)^

Nonetheless, the efficacy of tocolytic therapy remains controversial, especially with membrane rupture, due to limited data and its inability to consistently prevent preterm birth or reduce morbidity. Previous research lacks conclusive evidence supporting the effectiveness of short-term tocolysis in PROM cases. Consequently, further studies comparing the benefits and outcomes of tocolysis versus no treatment in similar clinical contexts are warranted.^(4,14)^

## Methods

The protocol for this systematic review and meta-analysis was prospectively registered in the International Prospective Register of Systematic Reviews (PROSPERO; registration number CRD420251043906). The review was conducted according to the recommendations of the Preferred Reporting Items for Systematic Reviews and Meta-Analyses (PRISMA 2020) statement.^(15)^

### Eligibility criteria

Studies were eligible for inclusion according to the following PICOTT criteria: Population: women with preterm prelabor rupture of membranes (PPROM); Intervention: tocolysis; Comparator: no tocolysis; Outcomes: neonatal mortality, neonatal intensive care unit admission, necrotizing enterocolitis, intraventricular hemorrhage, and neonatal sepsis; Type of study: randomized controlled trials and non-randomized comparative studies; and Time: no restrictions regarding publication date. Studies were excluded if they lacked an intervention or comparator group or included overlapping populations.

### Search strategy and data extraction

A systematic search was independently performed in MEDLINE, Embase, and the Cochrane Central Register of Controlled Trials (CENTRAL) for eligible studies published up to July 2024. The search strategy was developed using controlled vocabulary and free-text terms related to PPROM and tocolysis. The Medical Subject Headings (MeSH) terms included "Fetal Membranes, Premature Rupture" and "Tocolysis". Additional manual searches were performed by screening the reference lists of included studies and relevant systematic reviews to identify potentially eligible trials. The complete search strategy is available in the Supplementary Material (Table 1S).

Three independent reviewers assessed titles and abstracts identified through the search strategy. Potentially eligible articles underwent full-text assessment by the same reviewers. Disagreements during the selection process were resolved through discussion until consensus was achieved.

Data extraction was independently performed by two reviewers using a standardized Microsoft Excel spreadsheet. The extracted information included study characteristics, sample size, maternal baseline characteristics, gestational age at PPROM, details of the intervention and comparator groups, and reported maternal and neonatal outcomes. Any disagreements were resolved through consensus and, when necessary, discussion with a third reviewer.

### Risk of Bias and Quality Assessment

The risk of bias of the included randomized controlled trials was independently assessed by two reviewers using the Cochrane Risk of Bias 2 (RoB 2) tool. Disagreements were resolved by consensus after discussion with the first author. Studies were classified as having a low risk of bias, some concerns, or high risk of bias according to the overall judgment of the RoB 2 domains.^(16)^

The certainty of evidence was assessed using the Grading of Recommendations Assessment, Development and Evaluation (GRADE) approach. The quality of evidence was classified as high, moderate, low, or very low according to risk of bias, inconsistency, indirectness, imprecision, publication bias, and magnitude of treatment effects.

### Statistical Analysis

Data were analyzed using Cochrane Review Manager software (RevMan version 7.2.0). Dichotomous outcomes were summarized as odds ratios (ORs) with 95% confidence intervals (CIs). A random-effects model was used to account for expected clinical and methodological heterogeneity among studies. Statistical heterogeneity was assessed using Cochran's Q test and quantified using Higgins and Thompson's I^2^ statistic. The statistical significance of pooled estimates was determined using the Z test, and a p-value <0.05 was considered statistically significant.

## Results

### Study Selection and Characteristics

A total of 1,421 studies were screened. After duplicate removal and title screening, 12 studies underwent full-text assessment. Ultimately, the included studies comprised a total of 372 pregnant women, with 167 (44.89%) in the intervention group and 205 (55.10%) in the control group. All participants were followed until the end of pregnancy. Figure 1 illustrates the overall protocol for the study and provides details on the number of studies excluded. The mean maternal age across studies ranged from 26.6 to 33.4 years, and gestational age at inclusion varied between 24 and 34 weeks. The four included studies (5, 6, 7, 9) evaluated neonatal outcomes associated with the use or non-use of tocolytic therapy, specifically assessing rates of intraventricular hemorrhage, neonatal mortality, necrotizing enterocolitis, and neonatal sepsis. Only three (5,6,7) studies approached the ICU admission. Chart 1 summarizes the main characteristics of the included studies.

**Figure 1:**
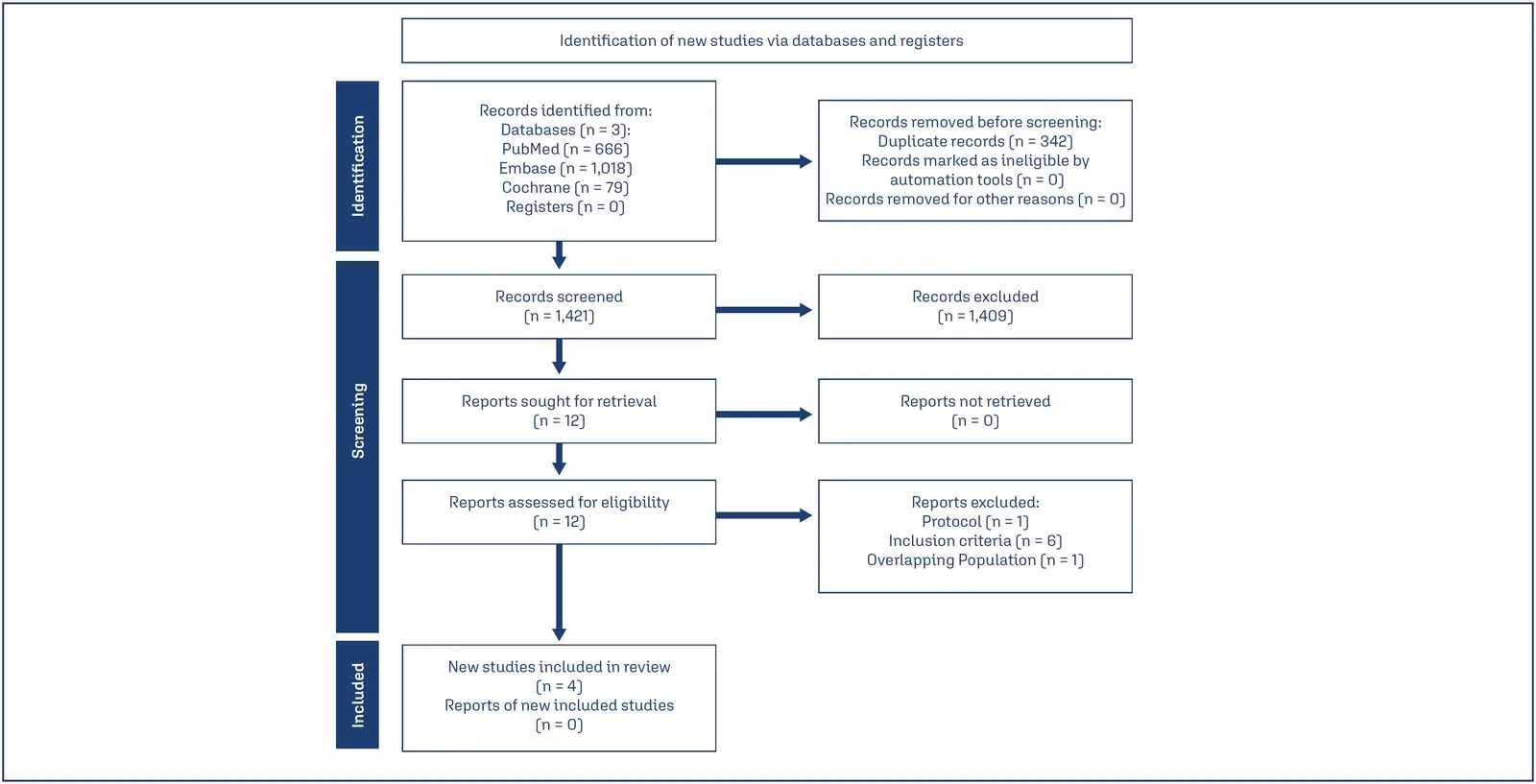
PRISMA flow diagram of study screening and selection

**Chart 1:** Baseline characteristics of included studies

Study	Population	Intervention	Control	N. of patients I/C	Maternal age† I/C	Nulliparous I/C
Nijman TA et al, 2016 ^(5)^	Women with PPROM without contractions between 24+0 and 33+6 weeks’ gestation.	Nifedipine	Placebo	25/25	33.4/32.9	16/14
Winden TSA et al, 2020 ^(6)^	Women with a singleton or multiple pregnancy and threatened preterm birth at 25+0 to 33+6 weeks’ gestation	Tocolysis	No tocolysis	46/46	33/33	26/25
Kulmala L et al, 2012 ^(7)^	Women with singleton pregnancies suffering from PPROM	Terbutaline	No tocolysis	61/102	29.1/26.6	23/46
Ehsanipoor RM et al, 2011^(9)^	Singleton pregnancies between 24° e 31°weeks with RM up to 24h rupture.	Indomethacin	Placebo	25/22	27.0/27.5	6/9

† Mean or median. C: control; I: intervention; PPROM: Preterm premature rupture of the membrane; RM: rupture of membranes.

### Pooled analysis of all studies

Compared to the control group, tocolysis did not result in significant differences in neonatal outcomes. The use of tocolytic therapy did not reduce neonatal mortality (OR: 2.02; 95% CI: 0.51–8.01; P = 0.32; I^2^ = 0%) or neonatal intensive care unit (NICU) admission (OR: 0.49; 95% CI: 0.02–14.57; P = 0.38; I^2^ = 85%) (Figure 2). Similarly, tocolysis did not reduce the incidence of necrotizing enterocolitis (OR: 1.35; 95% CI: 0.38–4.72; P = 0.64; I^2^ = 0%) or intraventricular hemorrhage (OR: 0.96; 95% CI: 0.01–138.63; P = 0.98; I^2^ = 58%) (Figure 3). Moreover, no significant difference was observed in the rate of neonatal sepsis (OR: 1.18; 95% CI: 0.25–5.54; P = 0.76; I^2^ = 68%) (Figure 1S).

**Figure 2:**
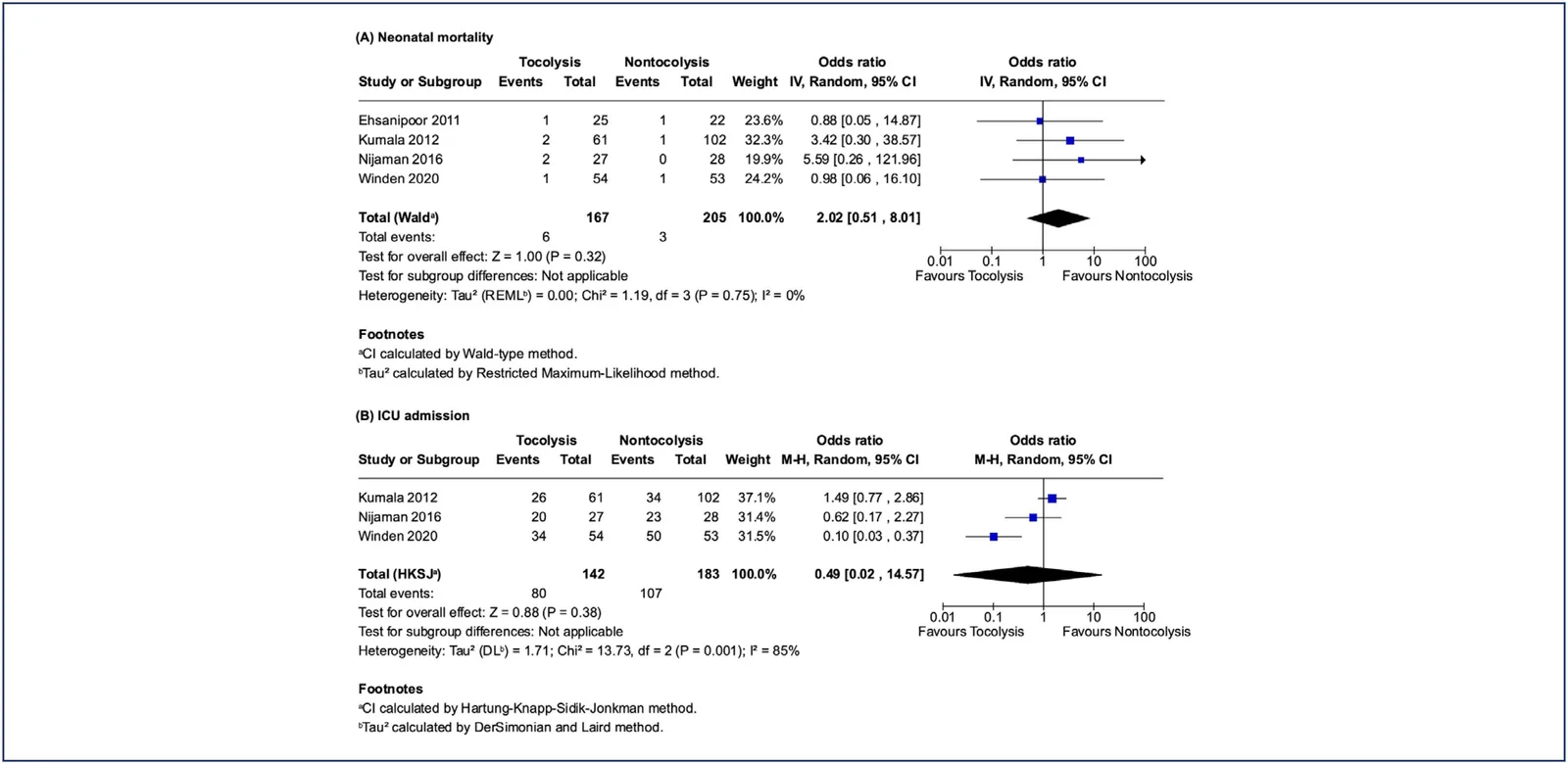
Forest plot of Studies Assessing Tocolytic Versus Non-Tocolytic Management in Preterm Premature Rupture of Membranes. (A): Neonatal mortality; (B): ICU admission

**Figure 3:**
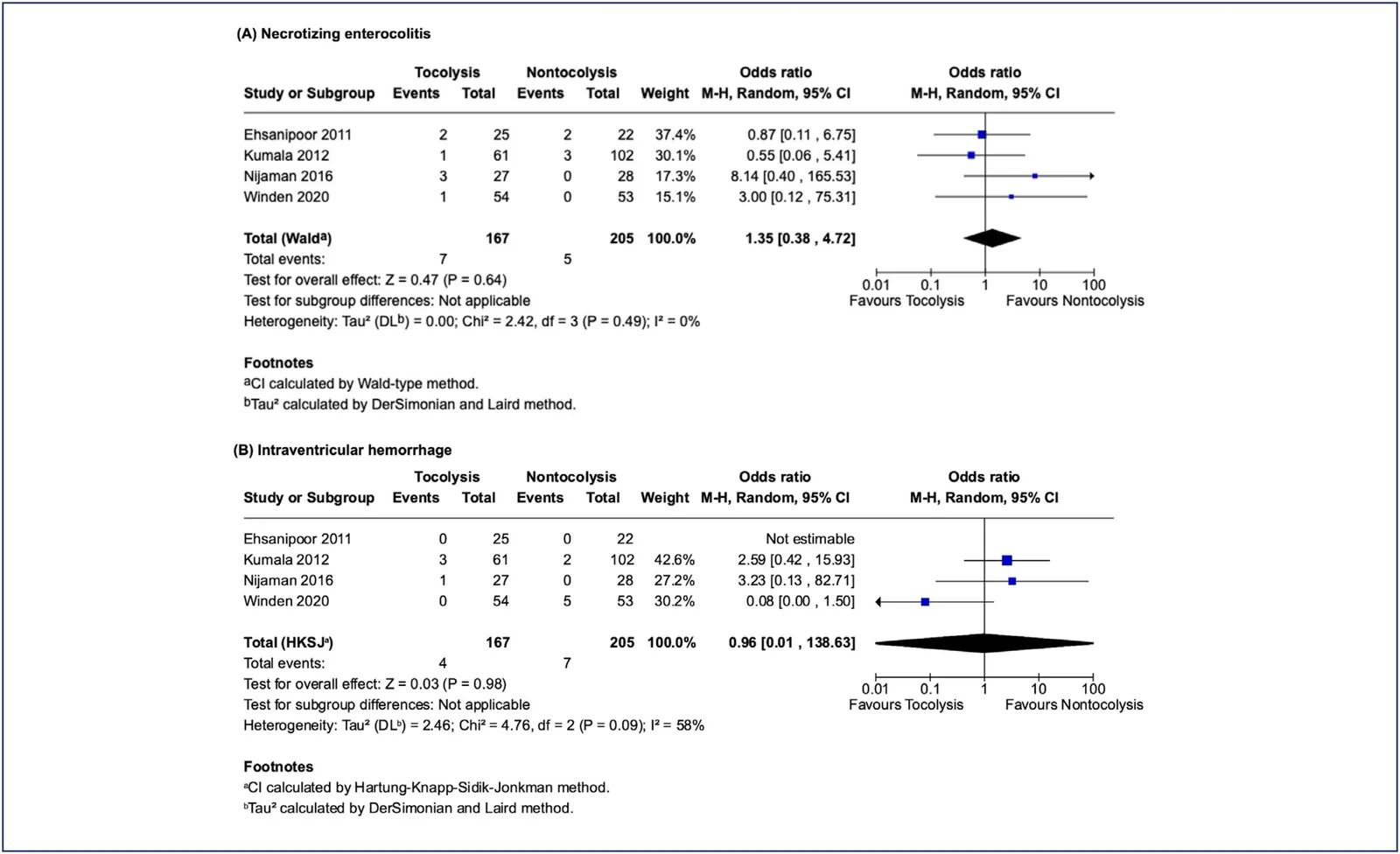
Forest plot of Studies Assessing Tocolytic Versus Non-Tocolytic Management in Preterm Premature Rupture of Membranes. (A): Necrotizing enterocolitis; (B) Intraventricular hemorrhage

### Risk of Bias and Quality of Evidence

The randomized controlled trials were assessed for risk of bias using the RoB 2 tool. Overall, all studies were classified as having some concerns regarding the risk of bias.^(16)^ Additionally, according to the ROBINS-I tool, all non-randomized studies demonstrated potential bias in at least one domain, mainly due to confounding factors and participant selection.^(17)^Figure S2 shows the individual risk-of-bias assessment for each study included in the meta-analysis. Based on the GRADE assessment, two outcomes, necrotizing enterocolitis and mortality, were classified as low-quality evidence, while sepsis was deemed to have very low-quality evidence. The primary factors contributing to the downgrading of evidence included result inconsistency due to heterogeneity, risk of bias related to moderate study limitations, and imprecision caused by wide confidence intervals (Figure 3S).

### Characteristics and findings of included studies

Four comparative studies evaluating the effect of tocolytic therapy in women with PPROM were included. The characteristics and main findings of each study are summarized below.

Nijman et al. (2016) conducted a randomized, placebo-controlled trial evaluating nifedipine as a tocolytic agent in women with PPROM. The study investigated the effects of nifedipine on perinatal outcomes compared with placebo. The authors did not demonstrate a significant improvement in neonatal outcomes with nifedipine use. Overall, the study did not provide evidence of a clear neonatal benefit associated with prophylactic tocolysis in women with PPROM.

Ehsanipoor et al. (2011) performed a randomized, double-masked, placebo-controlled trial assessing prophylactic indomethacin tocolysis in women with PPROM. The intervention was evaluated with respect to neonatal outcomes. The use of indomethacin did not significantly reduce adverse neonatal outcomes compared with placebo. The authors concluded that routine prophylactic indomethacin after PPROM did not provide a demonstrable neonatal benefit. Kulmala and Phupong (2012) evaluated terbutaline administration in women with PPROM in a randomized controlled trial. The study compared terbutaline with control management and assessed maternal and neonatal outcomes. The authors did not report relevant clinical benefits associated with prophylactic tocolysis.

Van Winden et al. (2020) investigated the association between tocolytic therapy and outcomes among women with threatened preterm birth and ruptured membranes using a propensity score analysis. Compared with no tocolysis, the use of tocolytic agents was not associated with clinically meaningful improvements in neonatal outcomes. Although the study included a broader population of women with ruptured membranes, its findings supported the lack of clear evidence for routine tocolytic therapy in this setting.

Overall, the included studies evaluated different tocolytic agents (nifedipine, indomethacin, and terbutaline) and used different methodological approaches. Despite these variations, none of the studies demonstrated a significant improvement in neonatal outcomes or consistent prolongation of pregnancy associated with tocolytic therapy compared with placebo or no tocolysis.

## Discussion

This systematic review and meta-analysis included four comparative studies, with a combined total of 372 patients, comprising two randomized controlled trials and two non-randomized studies, evaluating the effects of tocolytic therapy in women with PPROM. The meta-analysis demonstrated no statistically significant differences between the intervention and control groups regarding neonatal mortality (OR: 2.02; 95% CI: 0.51–8.01), ICU admission (OR: 0.49; 95% CI: 0.02–14.57), necrotizing enterocolitis (OR: 1.35; 95% CI: 0.38–4.72), intraventricular hemorrhage (OR: 0.96; 95% CI: 0.01–138.63), or neonatal sepsis (OR: 1.18; 95% CI: 0.25–5.54).

In the studies included in this review, the diagnosis of PPROM was primarily established through clinical assessment. Most trials confirmed membrane rupture by sterile speculum examination demonstrating amniotic fluid leakage from the cervical orifice, with additional confirmation by pooling, nitrazine, or ferning tests when applicable. Ultrasonography was also used in some studies to assess amniotic fluid volume and support the diagnosis when clinically indicated. Gestational age at presentation was an important baseline characteristic because of its influence on neonatal outcomes and clinical management. Overall, the diagnostic approaches used across the included studies were consistent with the clinical diagnosis of PPROM.

Many trials in that review were conducted more than three decades ago and did not uniformly use prophylactic antibiotics or antenatal corticosteroids, limiting their relevance to contemporary practice. The agreement between those historical data and the more recent randomized trials in our analysis reinforces the conclusion that, even with universal antibiotic and corticosteroid administration, tocolysis confers no clear neonatal advantage. However, the substantial heterogeneity observed for sepsis (I^2^ = 68 %) and NICU admission (I^2^ = 85 %) introduces considerable variability among studies and limits the confidence in this overall finding.^(18,19)^

Although tocolytic therapy has been proposed as a strategy to potentially improve clinical outcomes in women with PPROM, the findings of this review do not demonstrate a significant benefit in the evaluated neonatal outcomes. The lack of statistically significant differences in mortality, neonatal intensive care unit admission, necrotizing enterocolitis, intraventricular hemorrhage, and sepsis raises important questions regarding the routine use of tocolysis in the context of PPROM. While tocolysis may have an established role in selected cases of threatened preterm labor with intact membranes, its use following membrane rupture remains controversial because of the potential balance between theoretical benefits and maternal and neonatal risks. ^(20)^

Current international guidelines advise against routine tocolysis once membranes have ruptured, permitting at most a short course (≤ 48 h) to complete a steroid regimen or to facilitate maternal transfer to a tertiary center, provided there is no evidence of infection or placental abruption. The American College of Obstetricians and Gynecologists (ACOG) likewise does not routinely recommend tocolysis in PPROM, instead emphasizing individualized clinical judgment. Given the absence of proven neonatal benefit and the potential for increased maternal or fetal infection, the clinical use of tocolytic therapy should be restricted to these narrowly defined situations and discontinued once the objective is achieved. Conservative management with antibiotics and antenatal corticosteroids remains the evidence-based standard for PPROM, and the routine use of tocolytics should therefore be approached with caution.^(10,12,21,22)^

Our study presents several important limitations that should be acknowledged. Among the four studies included in this review, methodological limitations may compromise the robustness of the findings and reduce the strength of the evidence. Additionally, some studies exhibited potential biases related to the randomization process and participant selection, which may introduce systematic errors into the analysis. Another key limitation is the relatively small sample size observed across the included studies, which not only limits the statistical power but also reduces the generalizability of the findings. These concerns align with the broader assessment performed using the GRADE methodology, which classified the overall quality of evidence as low to very low for most of the evaluated outcomes. This grading was influenced by the presence of methodological bias, result inconsistency (heterogeneity), and imprecision, particularly reflected in the wide confidence intervals.

Taken together, these limitations highlight the need for further high-quality randomized controlled trials with larger, well-defined samples and standardized outcome measures. Future research should also aim to identify specific subgroups of patients with PPROM who might derive the greatest benefit from tocolytic therapy, considering factors such as gestational age, extent of membrane rupture, and presence of intrauterine infection.^(20,18,23)^

## Conclusion

In conclusion, the data presented in this meta-analysis suggest that tocolytic therapy in the management of preterm premature rupture of membranes (PPROM) does not provide a statistically significant improvement in major neonatal outcomes. No significant differences were observed between the tocolysis and control groups regarding rates of neonatal sepsis, mortality, ICU admission, necrotizing enterocolitis, or intraventricular hemorrhage. These findings, in conjunction with the overall low quality of available evidence, underscore the importance of individualized clinical decision-making and highlight the need to reconsider the routine use of tocolytics in this context. Given the lack of demonstrated benefit and the potential for risks, further high-quality, large-scale randomized controlled trials are essential to validate these findings and support the development of clear, evidence-based guidelines for the management of PPROM.

## Data Availability

The research data are described in the article presented.

## References

[1] Mendez-Figueroa H, Chauhan SP (2020). Tocolytic therapy in preterm premature rupture of membranes. Obstet Gynecol Clin North Am.

[2] Yamaji N, Suzuki H, Saito K, Swa T, Namba F, Vogel JP (2023). Tocolytic therapy inhibiting preterm birth in high-risk populations: a systematic review and meta-analysis. Children (Basel).

[3] Areia AL, Areia M, Mota-Pinto A (2020). Procalcitonin in preterm rupture of membranes: a systematic review and meta-analysis. Arch Gynecol Obstet.

[4] Mackeen AD, Seibel-Seamon J, Muhammad J, Baxter JK, Berghella V (2014). Tocolytics for preterm premature rupture of membranes. Cochrane Database Syst Rev.

[5] Nijman TA, van Vliet EO, Naaktgeboren CA, Oude Rengerink K, de Lange TS, Bax CJ (2016). Nifedipine versus placebo in the treatment of preterm prelabor rupture of membranes: a randomized controlled trial. Eur J Obstet Gynecol Reprod Biol.

[6] van Winden TM, Roos C, Nijman TA, Kleinrouweler CE, Olaru A, Mol BW (2020). Tocolysis compared with no tocolysis in women with threatened preterm birth and ruptured membranes: a propensity score analysis. Eur J Obstet Gynecol Reprod Biol.

[7] Kulmala L, Phupong V (2012). Effect of terbutaline on latency period in preterm premature rupture of membranes. Gynecol Obstet Invest.

[8] Anumba D, Soma-Pillay P, Bianchi A, Valencia González CM, Jacobbson B, FIGO Committee for Preterm Birth (2024). FIGO good practice recommendations on optimizing models of care for the prevention and mitigation of preterm birth. Int J Gynaecol Obstet.

[9] Ehsanipoor RM, Shrivastava V, Lee RM, Chan K, Galyean AM, Garite TJ (2011). A randomized, double-masked trial of prophylactic indomethacin tocolysis versus placebo in women with premature rupture of membranes. Am J Perinatol.

[10] (2020). Obstet Gynecol.

[11] Lorthe E, Marchand-Martin L, Letouzey M, Aubert AM, Pierrat V, Benhammou V (2024). Tocolysis after preterm prelabor rupture of membranes and 5-year outcomes: a population-based cohort study. Am J Obstet Gynecol.

[12] Ronzoni S, Boucoiran I, Yudin MH, Coolen J, Pylypjuk C, Melamed N (2022). Guideline No. 430: diagnosis and management of preterm prelabour rupture of membranes. J Obstet Gynaecol Can.

[13] Mu Y, Hu A, Kan H, Li Y, He Y, Fan W (2023). Preterm prelabor rupture of membranes linked to vaginal bacteriome of pregnant females in the early second trimester: a case-cohort design. Reprod Sci.

[14] Xiong Z, Pei S, Zhu Z (2022). Four kinds of tocolytic therapy for preterm delivery: systematic review and network meta-analysis. J Clin Pharm Ther.

[15] Page MJ, McKenzie JE, Bossuyt PM, Boutron I, Hoffmann TC, Mulrow CD (2021). The PRISMA 2020 statement: an updated guideline for reporting systematic reviews. BMJ.

[16] Sterne JA, Savović J, Page MJ, Elbers RG, Blencowe NS, Boutron I (2019). RoB 2: a revised tool for assessing risk of bias in randomised trials. BMJ.

[17] Sterne JA, Hernán MA, Reeves BC, Savović J, Berkman ND, Viswanathan M (2016). ROBINS-I: a tool for assessing risk of bias in non-randomised studies of interventions. BMJ.

[18] Lorthe E, Goffinet F, Marret S, Vayssiere C, Flamant C, Quere M (2017). Tocolysis after preterm premature rupture of membranes and neonatal outcome: a propensity-score analysis. Am J Obstet Gynecol.

[19] Ubom AE, Vatish M, Barnea ER (2023). FIGO Childbirth and Postpartum Hemorrhage Committee. FIGO good practice recommendations for preterm labor and preterm prelabor rupture of membranes: Prep-for-Labor triage to minimize risks and maximize favorable outcomes. Int J Gynaecol Obstet.

[20] World Health Organization (2022). WHO recommendation on tocolytic therapy for improving preterm birth outcomes.

[21] Lorthe E, Kayem G, TOCOPROM Study Group and the GROG (Groupe de Recherche en Obstétrique et Gynécologie) (2021). Tocolysis in the management of preterm prelabor rupture of membranes at 22-33 weeks of gestation: study protocol for a multicenter, double-blind, randomized controlled trial comparing nifedipine with placebo (TOCOPROM). BMC Pregnancy Childbirth.

[22] Enengl S, Rath W, Kehl S, Oppelt P, Mayr A, Stroemer A (2024). Differences between Current Clinical Practice and Evidence-Based Guideline Recommendations Regarding Tocolysis - an Austria-wide Survey. Geburtshilfe Frauenheilkd.

[23] Haddaway NR, Page MJ, Pritchard CC, McGuinness LA (2022). PRISMA2020: an R Package and Shiny App for Producing PRISMA 2020-compliant flow diagrams, with interactivity for optimised digital transparency and open synthesis. Campbell Syst Rev.

