# Supplementary material for: Tocolytic therapy for preterm premature rupture of membranes: a systematic review and meta-analysis
Source: Rev Bras Ginecol Obstet. 2026 Sep 23;48:e-rbgo33. doi: 10.61622/rbgo/2026rbgo33 (PMC13690392; doi:10.61622/rbgo/2026rbgo33)
Supplement: Supplementary file 1 [file 1806-9339-rbgo-48-e-rbgo33-Supp01.pdf]

Supplementary material

Table 1S. Search strategy

Data base	Search strategy	Results
PubMed	(“membrane premature ruptures” OR PROM OR “fetal membranes, premature rupture”[mh] OR “preterm prelabor rupture of membranes” OR “premature rupture of fetal membranes” OR “membrane premature rupture” OR “preterm premature rupture of fetal membranes”) AND (“tocolysis”[mh] OR tocolytic OR indomethacin OR “cyclooxygenase inhibitors” OR nifedipine OR terbutaline OR “beta-agonists” OR ritodrine OR atosiban)	666*
Cochrane	(“membrane premature ruptures” OR PROM OR “fetal membranes, premature rupture”[mh] OR “preterm prelabor rupture of membranes” OR “premature rupture of fetal membranes” OR “membrane premature rupture” OR “preterm premature rupture of fetal membranes”) AND (“tocolysis”[mh] OR tocolytic OR indomethacin OR “cyclooxygenase inhibitors” OR nifedipine OR terbutaline OR “beta-agonists” OR ritodrine OR atosiban)	79*
EMBASE	(‘membrane premature ruptures’ OR prom OR ‘fetal membranes, premature rupture’/exp OR ‘fetal membranes, premature rupture’ OR ‘premature rupture of fetal membranes’/exp OR ‘premature rupture of fetal membranes’ OR ‘membrane premature rupture’/exp OR ‘membrane premature rupture’ OR ‘preterm premature rupture of fetal membranes’) AND (‘tocolysis’/exp OR ‘tocolysis’ OR tocolytic OR ‘nifedipine’/exp OR ‘terbutaline’/exp OR ‘atosiban’/exp OR atosiban)	1018*

* Complete electronic search strategies for each database in July 2024

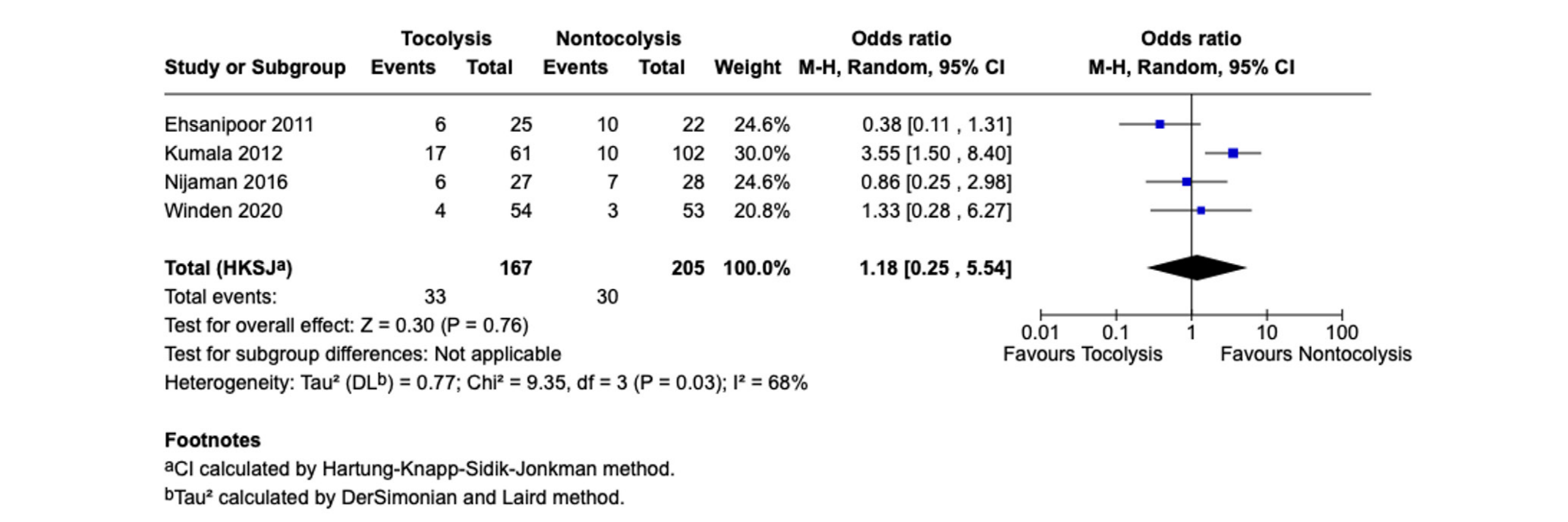

Figure 1S. Forest plot of neonatal sepsis

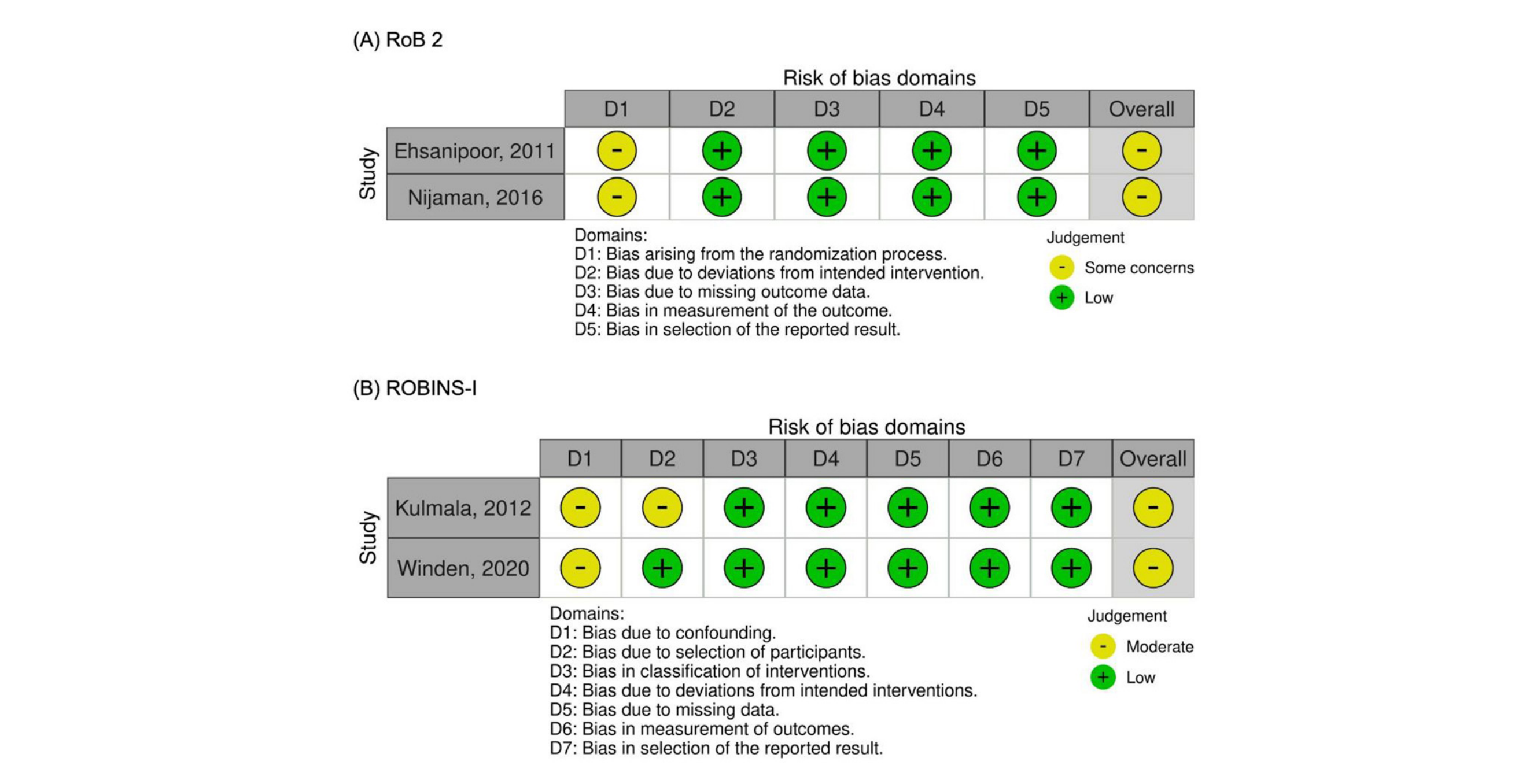

Figure 2S. Critical appraisal of individual studies according to the Cochrane Collaboration’s tool for assessing risk of bias in randomized and non-randomized studies. (A) RoB 2; (B) ROBINS-I

Tocolysis compared to non tocolysis for preterm premature rupture of membranes

Patient or population: preterm premature rupture of membranes
Setting:
Intervention: Tocolysis
Comparison: non tocolysis

Outcomes	№ of participants (studies) Follow-up	Certainty of the evidence (GRADE)	Relative effect (95% CI)	Anticipated absolute effects	
				Risk with non tocolysis	Risk difference with Tocolysis
Sepsis	372 (4 non-randomised studies)	⊕◯◯◯ Very low[a,b,c]	**OR 1.18** (0.25 to 5.54)	146 per 1.000	**22 more per 1.000** (105 fewer to 341 more)
Necrotizing enterocolitis	372 (4 non-randomised studies)	⊕⊕◯◯ Low[a,c]	**OR 1.35** (0.38 to 4.72)	24 per 1.000	**8 more per 1.000** (15 fewer to 81 more)
Mortality	372 (4 non-randomised studies)	⊕⊕◯◯ Low[a,c]	**OR 2.02** (0.51 to 8.01)	15 per 1.000	**14 more per 1.000** (7 fewer to 92 more)

***The risk in the intervention group** (and its 95% confidence interval) is based on the assumed risk in the comparison group and the **relative effect** of the intervention (and its 95% CI).

CI: confidence interval; **OR:** odds ratio

GRADE Working Group grades of evidence
High certainty: we are very confident that the true effect lies close to that of the estimate of the effect.
Moderate certainty: we are moderately confident in the effect estimate: the true effect is likely to be close to the estimate of the effect, but there is a possibility that it is substantially different.
Low certainty: our confidence in the effect estimate is limited: the true effect may be substantially different from the estimate of the effect.
Very low certainty: we have very little confidence in the effect estimate: the true effect is likely to be substantially different from the estimate of effect.

Explanations

a. Studies with moderate risk of bias. Downgraded by one level for risk of bias.
b. High heterogeneity (I2=68%). Downgraded by one level for inconsistency
c. Wde confidence interval. Downgraded by one level for imprecision.

Figure 3S. GRADE assessment
